# Gelsolin Facilitates Estrogen Receptor Beta Nuclear Translocation and Transcriptional Repression of Genes Associated with Alzheimer Disease

**DOI:** 10.3390/receptors4020010

**Published:** 2025-05-01

**Authors:** Yoldas Yildiz, Angela H. S. Fan, Amanda A. Hartoun, Sarah Flury, Yan Ngai, Toni R. Pak

**Affiliations:** Stritch School of Medicine, Loyola University Chicago, Maywood, IL 60153, USA

**Keywords:** ESR2, cytoskeleton, nuclear receptor, aging, brain, neuron, APP, ITPKB

## Abstract

**Background/Objectives:**

Gelsolin (GSN) is an actin-binding protein that helps maintain neuronal structure and shape, regulates neuronal growth, and apoptosis. Our previous work demonstrated that GSN associated with estrogen receptor beta (ERβ1) in the brains of female rats, but this association was lost in advanced age. GSN was also required for ERβ1-mediated transcriptional repression at activator protein-1 (AP-1) motifs upstream of a minimal gene promoter. However, the consequences of the loss of GSN:ERβ1 protein interaction on ERβ1 nuclear translocation and transcriptional repression at AP-1 sites located within complex endogenous gene promoters remained unclear.

**Methods:**

We used immunofluorescent super resolution microscopy and luciferase reporter assays to test the hypothesis that GSN facilitates ERβ1 nuclear translocation and transcriptional repression of two genes relevant for Alzheimer Disease: APP (amyloid-beta precursor protein) and ITPKB (inositol-1,4,5-trisphosphate 3-kinase B).

**Results:**

Our results revealed the novel finding that GSN is required for ERβ1 ligand-independent nuclear translocation in neuronal cells. Moreover, we show that GSN increased APP and ITPKB promoter activity, which was repressed by ERβ1.

**Conclusions:**

Together, these data revealed the importance of the cytoskeletal protein, GSN, in regulating intracellular trafficking of nuclear receptors and demonstrate the first evidence of ERβ1 directly regulating two genes that are implicated in the progression of AD.

## 1. Introduction

Estrogen receptors (ER)α and ERβ1 are the main mediators of the biological effects of estrogens. These high-affinity receptors are encoded by two distinct genes, ESR1 and ESR2, respectively, and activate or repress gene transcription upon ligand binding [1]. Both receptors can interact directly with DNA by binding to specific motifs (e.g., estrogen response elements; ERE) within gene promoters, or tether to DNA as part of a multiprotein complex with other transcription factors [2,3]. However, ERβ1 is a relatively weak transcriptional activator at EREs compared to ERα, and genome-wide mapping has revealed that ERβ1 is preferentially tethered at AP-1 (activator protein-1) sites [4]. The full array of cellular mechanisms regulating ERβ1 nuclear translocation and binding to other transcription factors has not been determined.

Notably, ERβ1 is expressed at higher levels in the brain post-menopause compared to ERα and has many biological effects unrelated to reproduction, such as cardio- and neuroprotection [5-10]. Our previous study showed that ERβ1 protein–protein interactions are altered in the brain with age and 17β-estradiol (E2) treatment in a rat model of menopause [11]. Specifically, E2 treatment increased ERβ1 association with the actin-binding protein gelsolin (GSN), but this association was lost in aged animals. Moreover, GSN knockdown abolished ERβ1-induced repression of AP-1-mediated, but not ERE-mediated, promoter activity [11]. Gelsolin has also been shown to facilitate translocation and transcriptional regulatory ability of the androgen receptor, another nuclear steroid receptor [12]. Taken together, these results raised the possibility that GSN facilitates ERβ1 nuclear translocation and tethering to transcription factors that bind AP-1 sites.

Gelsolin is important for maintaining neuronal cytoskeleton organization, cell motility and shape. Notably, GSN can dissolve actin fibrils through its role in transforming filamentous actin to monomeric actin, a feature that also allows it to dissolve plaque forming amyloid-β fibers [13], such as those that accumulate pathologically in Alzheimer Disease (AD). A comprehensive analysis of differentially expressed genes in AD patients identified genes that had increased expression levels correlated with disease severity [14]. Two such genes, the amyloid-beta precursor protein (APP) and the inositol-trisphosphate 3-kinase B (ITPKB), have multiple AP–1 sites and their dysregulation has been strongly implicated in AD progression [15,16]. The processing of the APP leads to the accumulation of amyloid β plaques, a significant hallmark of AD. Human AD patient samples also have increased expression of ITPKB, which, when replicated in cell models, shows increased cell death and amyloid β production, suggesting that ITPKB can serve as an important regulator of neuronal cell apoptosis and APP processing [16]. Importantly, many human gene promoters contain at least one AP-1 site with 11% containing two and 4% containing more than two [17]. These data underscore the translational significance of understanding the molecular mechanism regulating ERβ1-mediated repression at AP-1 sites.

The role of GSN in regulating ERβ1 nuclear translocation and transcriptional repression at AP-1 sites remains unclear. Therefore, in this study, we hypothesized that GSN facilitates ERβ1 translocation to the nucleus in neuronal cells. In addition, we hypothesized that ERβ1 represses human APP and ITPKB gene transcription, and that the repression is facilitated by GSN. Our results demonstrate the novel finding that GSN is required for ligand-independent translocation of ERβ1 and increases APP and ITPKB promoter activity. Further, we show that ERβ1 represses both APP and ITPKB gene promoters independently of GSN and ligand. Together, these data reveal the importance of cytoskeletal proteins in regulating intracellular trafficking of nuclear receptors and demonstrate the first evidence of ERβ1 directly regulating two genes implicated in the progression of AD.

## 2. Materials and Methods

### 2.1. Cell Culture

Human neuroblastoma cell lines (SK-N-SH), which were isolated from a four-year-old female, were purchased from American Type Culture Collection (ATCC, Manassas, VA, USA) and maintained in the recommended normal growth media (minimal essential media with Earle’s balanced salts (MEM/EBSS—Cytiva, MA, USA. Cat#SH30244.01) plus 1% L-glutamine and 10% fetal bovine serum (FBS)). Cells were grown to 60–70% confluency prior to all experiments. All experiments were performed within 10 passages, and a different passage was used to represent each biological replicate. SK-N-SH cells were validated for the endogenous expression of GSN (positive signal by Western blot) and ERβ1 (negative signal by mass spectrometry, Figure S1).

### 2.2. Cell Treatments

Normal growth media (described above) was replaced with media containing phenolred-free MEM/EBSS plus 1% L-glutamine and 10% charcoal/dextran stripped FBS (GeminiBio, Sacramento, CA, USA. Cat #100-106-500) for 48 h to eliminate all sources of hormones within the media. Cells were treated with 10 nM 17β-estradiol (E2; Steraloids) or an equivalent volume of media containing 0.001% ethanol (vehicle control) for 15 h.

### 2.3. Plasmid Constructs

The plasmid expression vector containing an insert for human ERβ1 was generously provided by Dr. Shuk-Mei Ho (University of Cincinnati, Cincinnati, OH, USA) and has been extensively characterized [18]. The plasmid vector containing the insert for rat ERβ1Δ3 was a generous gift, and was characterized by Dr. Robert J. Handa [19]. ERβ1Δ3 is full length ERβ1 (AA1-530) with an in-frame loss of exon 3 (117 nucleotides; 39 AA) in the DNA binding domain, which includes the second zinc finger. GSN siRNA and scrambled siRNA were purchased from Santa Cruz Biotech, Inc, Dallas, TX, USA. Reporter constructs for human APP (GoClone cat #S719381), human ITPKB (GoClone cat #S701052), human HPRT (GoClone cat #S705060) and an empty promoter-less plasmid (GoClone cat #S790005) were purchased from SwitchGear Genomics, Menlo Park, CA, USA. Sequences for all plasmids were verified by DNA sequencing (ACGT, Inc., Wheeling, IL, USA).

### 2.4. Transient Transfections

All constructs were transfected in replicate wells of 6/construct/condition within each assay, and each assay was repeated 4–6 times using separate passages. Transfections were performed using a lipid-mediated transfection reagent (Fugene LLC, Middleton, WI, USA, cat. # SI-1000). Endogenous expression of GSN in SK-N-SH cells was reduced using lipid-mediated transient transfection of 70 nM siRNA against GSN, or scrambled sequence siRNA as a control. Expression vectors and reporter constructs were transfected at a ratio of 150 ng:1.0 μL (DNA:Fugene). Total plasmid DNA was kept equal for all wells/conditions.

### 2.5. Luciferase Assays

SK-N-SH cells were seeded at 5000 cells/well in white 96-well plates with clear bottoms. 24 h after seeding, cells were co-transfected with expression vectors and/or reporter constructs as appropriate for each experiment. Equivalent total DNA concentrations were used in each well for every experiment. 48 h after transfection, plates were stored in −80 °C for at least 6 h. Plates were thawed on wet ice before the addition of LightSwitch Assay Reagent per manufacturer instructions (LightSwitch, cat #LS100), and luciferase activity was measured at 480 nm using a CLARIOstar multimode microplate reader (BMG Labtech, Cary, NC, USA).

### 2.6. Western Blot

SK-N-SH cells were plated at a density of 1 × 10^6^ cells/plate in 10 cm diameter cell culture plates and transfected as described above. M-PER^™^ Mammalian Protein Extraction Reagent (500 μL; Thermo Scientific^™^, Cat. 78503) was added to each 10 cm diameter cell culture plate and stored in −80 °C freezer to lyse cells. Adherent cells were harvested using a scraper tool, vortexed, and the resulting protein supernatant was transferred to microcentrifuge tubes. Total protein was quantified using a Pierce^™^ BCA (bicinchoninic acid) assay kit according to the manufacturer instructions (Thermo Scientific; Cat. 23225). Next, total proteins (30 mg/sample) were separated by mass using electrophoresis on 12% SDS PAGE gels and then transferred onto a PVDF membrane (Thermo Scientific, Cat # 88518) for 1 h at 100 V. The amount of protein per sample needed for GSN antibody was calculated using a calibration curve. To normalize target signals, a total protein stain was performed according to the manufacturer’s protocol (LI-COR Revert^™^ 700 Total Protein Stain Kit). The PVDF membrane was blocked with 5% milk in TBS-T for 1 h and then incubated with mouse GSN primary antibody (Santa Cruz, Cat #sc-514502) overnight at 4 °C. Following incubation, the PVDF membrane was washed 3 × 10 min in 10 mL of 5% milk in TBS-T. Then, the membrane was incubated in donkey anti-mouse secondary antibody (LI-COR IRDye^®^ 800CW P/N: 926-32212) for 1 h at room temperature, and washed 3× for 10 min in TBS. The PVDF membrane was then imaged using Azure Sapphire FL Biomolecular Imager (RGBNIR, Dublin, CA, USA) according to the manufacturer instructions. Gelsolin protein levels were quantified using AzureSpot Pro software, v1.2-450 (Azure Biosystems, Dublin, CA, USA).

### 2.7. Immunofluorescence

Cells were plated into 6-well plates at a density of 200,000 cells/well and transfected as described above. SK-N-SH cells were washed with 1 × PBS and fixed in 4% paraformaldehyde for 20 min. Cells were then permeabilized with 1% Triton-X in Tris-buffered Saline (TBS–T) for 10 min and washed with TBS 3 × 5 min. Nonspecific antigens were blocked with blocking buffer (10% normal goat serum in 1% TBS–T), for 1 h at room temperature, followed by incubation of primary mouse GSN monoclonal antibody (Santa Cruz, cat #sc-514505) at 1:250 in blocking buffer overnight at 4 °C. The ERβ1 expression vector was modified by adding a sequence for enhanced green fluorescent protein (eGFP) at the C-terminus enabling microscopic visualization without the need for antibodies against ERβ1. Total sequence was verified by DNA sequencing (ACGT Inc., Wheeling, IL, USA). Cells were then washed with TBS 3 × 5 min at room temperature, then incubated with secondary goat anti-mouse Alexa Fluor 647 (ThermoFisher Scientific Cat #A32728) at 1:1000 in blocking buffer for 2 h at room temperature followed by another round of washing with TBS 3 × 5 min. A drop of prolong diamond antifade mountant with DAPI (ThermoFisher Scientific, cat #P36962) was applied to coverslips and allowed to cure on microscope slides at room temperature for one hour. Slides were then sealed with nail polish and stored at −20 °C until imaging.

### 2.8. Imaging

Immunostained fixed cells were imaged on a Marianas Imaging System (Intelligent Imaging Innovations, Denver, CO, USA) consisting of an Axio Observer 7 (Zeiss, Oberkochen, Germany) microscope base, a CSU-W1 SoRa Spinning Disk (Yokogawa, Sugar Land, TX, USA), and a laser launch that included 405, 488, and 637 nm lasers and Slidebook software, version 2024.2 (Intelligent Imaging Innovations, CO, USA). Images were collected using a Plan-Apochromat 63X/1.46 NA oil TIRF objective, using the SoRa 1× and 2.8× multiplier on a Prime 95B sCMOS (Photometrics, Tucson, AZ, USA) camera. Total cell fluorescence was measured by outlining individual cells in ImageJ (version 1.53t, NIH, Bethesda, MD, USA) and multiplying the mean absorbance with total size of cell. Nuclear fluorescence was measured by outlining the nucleus of each individual cell in ImageJ and multiplying the mean absorbance with nuclear size. Background fluorescence was calculated by measuring a background region outside of cells and multiplying the size of the region by the mean fluorescence. The calculated background fluorescence was subtracted from the calculated total cell fluorescence and nuclear fluorescence. Cytoplasmic fluorescence was calculated by subtracting the calculated nuclear fluorescence from the total cell fluorescence.

### 2.9. Statistics

Statistical analyses were performed using PRISM software (v10.4.0, San Diego, CA, USA). All groups passed normality using homoscedasticity tests. Experimenters were blinded to treatments during data analysis. Data were analyzed by one-way and two-way ANOVA unless otherwise noted in figure captions. Data are displayed as mean ± SEM and statistical significance was determined at *p* < 0.05.

## 3. Results

### 3.1. Gelsolin Is Required for Nuclear Translocation of ERβ1

**Figure 1.**
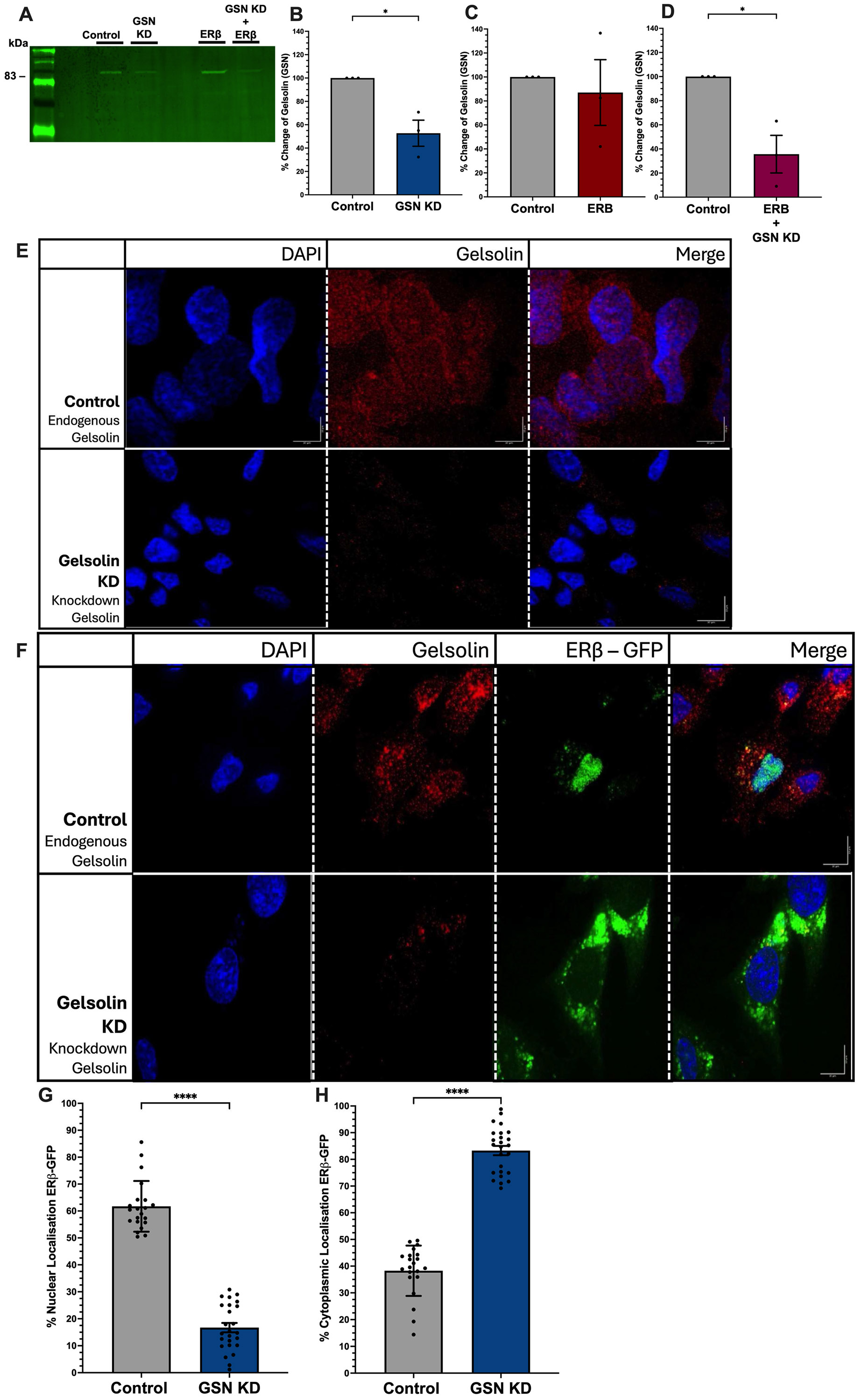
Gelsolin (GSN) is required for nuclear translocation of ERβ1. (**A–D**) Representative Western blot and quantification depicting GSN expression in SK-N-SH cells with control (scrambled siRNA) and siRNA-GSN knockdown, and ±co-transfection with ERβ1-eGFP. (**E**) Immunofluoresent images representing endogenous GSN expression in SK-N-SH cells with control (scrambled siRNA) and siRNA-mediated GSN knockdown, and (**F**) ±ERβ1-eGFP co-transfection. Quantification of nuclear (**G**) and cytoplasmic (**H**) ERβ1-eGFP. Each data point represents one cell, with at least 20 cells across three biological replicates. Green = ERβ1-eGFP. Blue = DAPI. Red = Gelsolin. Data are depicted as mean ± SEM. An * denotes *p* < 0.05; ****, *p* < 0.0001. Scale bar = 10 μm.

Previous studies showed that GSN was required for facilitating nuclear import of the androgen receptor [12], which is structurally similar to ERβ1. To test if GSN is required for nuclear translocation of ERβ1, we co-transfected a neuronal cell line (SK-N-H cells) with an ERβ1-eGFP expression vector and GSN-siRNA, followed by immunofluorescent quantification of the ERβ1 nuclear–cytoplasmic ratio. First, we confirmed the absence of ERβ1 by mass spectrometry and the expression of endogenous GSN by Western blot (Supplemental Data: https://10.6084/m9.figshare.28459037 accessed on 2 March 2026). Endogenous GSN expression is seen throughout the cytoplasm and nucleus in SK–N–SH cells and siRNA-mediated knockdown of GSN resulted in an approximate 50% reduction in GSN protein levels, as confirmed through Western blot analysis (Figure 1A,B,E). Our previous study demonstrated that this level of knockdown was sufficient to impair ERβ1-mediated repression at an AP-1 site and that depleting GSN by more than 50% caused cell death [11]. Gelsolin was significantly reduced throughout the whole cell with only a few puncta visible in the cytoplasm (Figure 1E). Importantly, transient transfection with ERβ1-eGFP did not alter the endogenous levels of GSN (Figure 1A,C), and more than half of ERβ1-eGFP was localized in the nucleus (Figure 1F—top row). Quantification of nuclear and cytoplasmic proportions of ERβ1 show a ~60% to 40% ratio, respectively, in the presence of endogenous GSN (Figure 1G,H). Strikingly, these proportions of nuclear and cytoplasmic ERβ1 shift when GSN was depleted through siRNA-mediated knockdown, resulting in less than 20% of ERβ1-eGFP being localized to the nucleus (Figure 1G) and over 80% in the cytoplasm (Figure 1H). These results demonstrate a key role for GSN to facilitate nuclear localization of ERβ1, without which a larger portion of ERβ1 would stay sequestered in the cytoplasm.

### 3.2. 17β-Estradiol Induces ERβ1 Nuclear Translocation in the Absence of GSN

**Figure 2.**
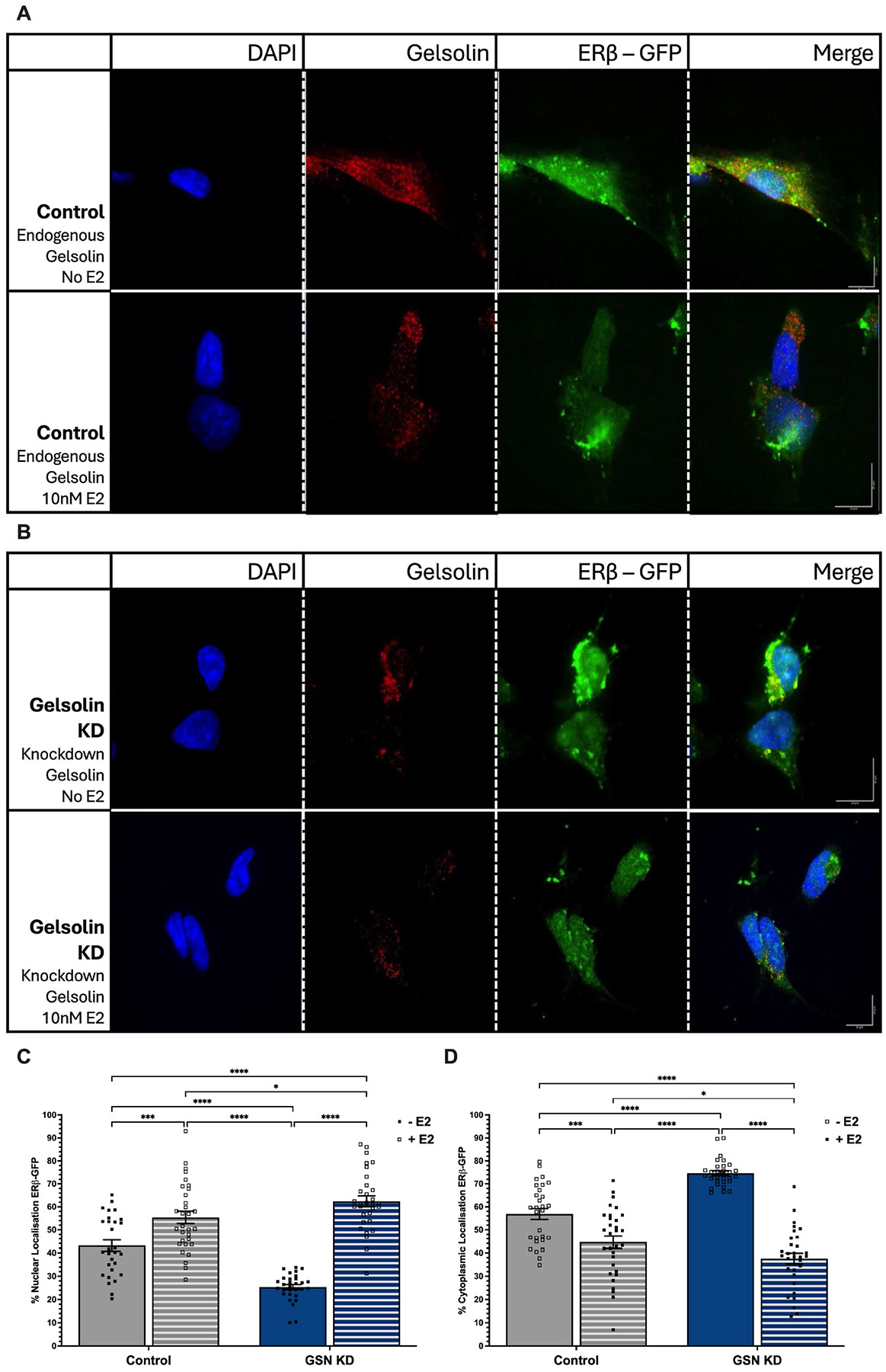
17β-estradiol (E2) induces ERβ1 nuclear translocation in the absence of GSN. (**A**) Immunofluoresent images representing endogenous GSN expression in SK-N-SH cells with control (scrambled siRNA) and siRNA-GSN knockdown ±10 nM E2, and (**B**) ±cotransfection with ERβ1-eGFP. Quantification of nuclear (**C**) and cytoplasmic (**D**) ERβ1-eGFP. Each data point represents one cell, with at least 20 cells across three biological replicates. Green = ERβ1-GFP. Blue = DAPI. Red = Gelsolin. Data are depicted as mean ± SEM. An * denotes *p* < 0.05, *** *p* < 0.001, and **** *p* < 0.0001. Scale bar = 10 μm.

Estrogen receptors are classified as “ligand-activated” transcription factors. Ligand binding to ERβ1 and ERα induces a conformational change that allows for dynamic changes in protein: protein interactions. Therefore, we next investigated whether GSN-mediated nuclear localization of ERβ1 was altered in response to E2 treatment. Cells were transfected as described above and then treated with 10 nM E2 for 3 h prior to imaging. We quantified the ERβ1 cytoplasmic: nuclear ratio in the presence of normal endogenous GSN levels. Similarly to the results shown in Figure 1, the cytoplasmic–nuclear ratio of ERβ1 was higher (60:40) in the absence of E2 prior to GSN knockdown (Figure 2A—top row,C,D); however, this ratio was reversed (40:60) in response to E2 treatment (Figure 2A—bottom row,C,D). These results demonstrate that E2 significantly increased ERβ1 nuclear localization in cells with normal endogenous levels of GSN. Unexpectedly, we also observed a shift in endogenous GSN distribution in the presence of E2. Treatment resulted in GSN clustered in a perinuclear pattern compared to being widely dispersed throughout the cell in the untreated cells (Figure 2A). The cytoplasmic–nuclear ratio of ERβ1 after E2 treatment was quantified following GSN siRNA knockdown. Similarly to the results shown in Figure 1, GSN knockdown resulted in a predominant sequestering of ERβ1 in the cytoplasm (70%) in the absence of E2 treatment (Figure 2B—top row,C,D). However, in contrast to GSN knockdown alone, ERβ1 nuclear translocation was restored following E2 treatment (40:60) (Figure 2B—bottom row,C,D).

### 3.3. ERβ1 Represses APP and ITPKB Promoter Activity

**Figure 3.**
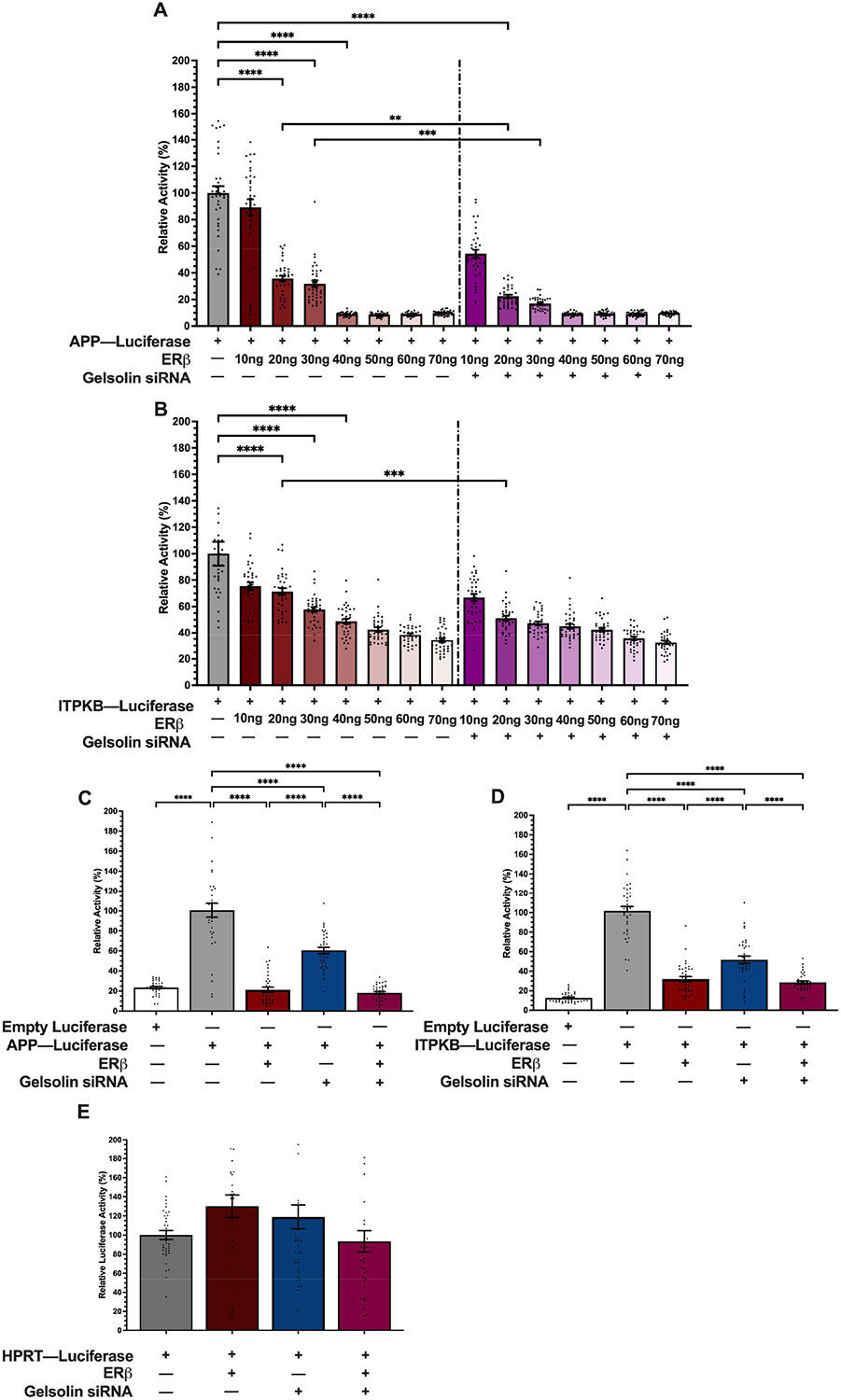
ERβ1 represses APP and ITPKB promoter activity. Luciferase activity was measured in SK-N-SH cells following transfection with human promoters for APP-luc (**A**) and ITPKB-luc (**B**) and varying concentrations of ERβ1 ± siRNA-mediated GSN knockdown. (**C–E**) Luciferase was measured in SK-N-SH cells following transfection with human promoters for APP-luc (**C**) and ITPKB-luc (**D**), or HPRT-luc (**E**) and 20 ng ERβ1 ± siRNA-mediated GSN knockdown. Relative promoter activity was calculated based on percentage (%) change from promoter-only group. Data sets are from 3 independent biological replicates with 12 technical replicates each. Data are depicted as mean ± SEM. An ** denotes *p* < 0.01, ***, *p* < 0.001, **** *p* < 0.0001.

Our previous work demonstrated that GSN knockdown abolished ERβ1-induced repression at AP-1 motifs located upstream of a minimal thymidine kinase promoter but had no effect on ERβ1 activation at an ERE [11]. However, it is unknown if GSN knockdown has a similar effect on ERβ1-mediated activity for complex gene promotors that harbor AP-1 motifs. Therefore, we tested if ERβ1 repressed the human APP and/or ITPKB promoters, which are correlated with increased AD severity and contain multiple AP-1 motifs [15,16]. SK-N-SH were co-transfected with human APP-luc or ITPKB-luc promoters and increasing concentrations of an ERβ1 expression vector. ERβ1 significantly repressed the APP promoter in a concentration-dependent manner starting as low as 20 ng plasmid DNA (Figure 3A). Maximum repression of the APP promoter was achieved with 40 ng plasmid DNA, where the promoter activity was repressed by more than 90%. ERβ1 also repressed the ITPKB promoter in a concentration-dependent manner, with a 20% reduction observed with 10 ng plasmid DNA, and reaching a maximum 70% repression with 70 ng plasmid DNA (Figure 3B). Next, we tested if ERβ1 repression of APP and ITPKB was dependent on GSN. GSN-siRNA knockdown further repressed ERβ1-mediated repression of both APP and ITPKB promoters (Figure 3A,B) but this additive effect was only observed with 20 ng concentrations of ERβ1 plasmid DNA. Therefore, 20 ng of the ERβ1 expression vector was used for all of the following experiments.

### 3.4. GSN Enhances APP and ITPKB Promoter Activity

The results shown in Figure 3 demonstrated that ERβ1 repression could be enhanced by knocking down GSN. However, to our knowledge, there are no previous reports of GSN modulating APP and/or ITPKB promoter activity or gene expression. Therefore, we tested the effects of GSN knockdown alone on promoter activity. Transient transfection of both the APP and ITPKB promoter luciferase constructs yielded a significant increase in luciferase activity compared to the empty promoter-less luciferase construct, demonstrating that the human neuroblastoma SK-N-SH cells have the constellation of endogenous transcription factors required for their expression (Figure 3C,D). Consistent with the results in Figure 3A,B, the addition of ERβ1 significantly repressed APP promoter activity by 80% and ITPKB promoter activity by 70%. Unexpectedly, siRNA-mediated knockdown of GSN alone (i.e., in the absence of ERβ1) also decreased the activity of both promoters (APP—60%, ITPKB—55%), suggesting that endogenous levels of GSN are required for normal expression of these genes. Importantly, neither ERβ1 nor GSN-siRNA had any effect on hypoxanthine phosphoribosyltransferase 1 (HPRT) promoter activity (Figure 3E), demonstrating that the observed effects of GSN and ERβ1 are specific to APP and ITPKB.

### 3.5. ERβ1 and GSN Regulation of APP and ITPKB Promoters Is Independent of E2

**Figure 4.**
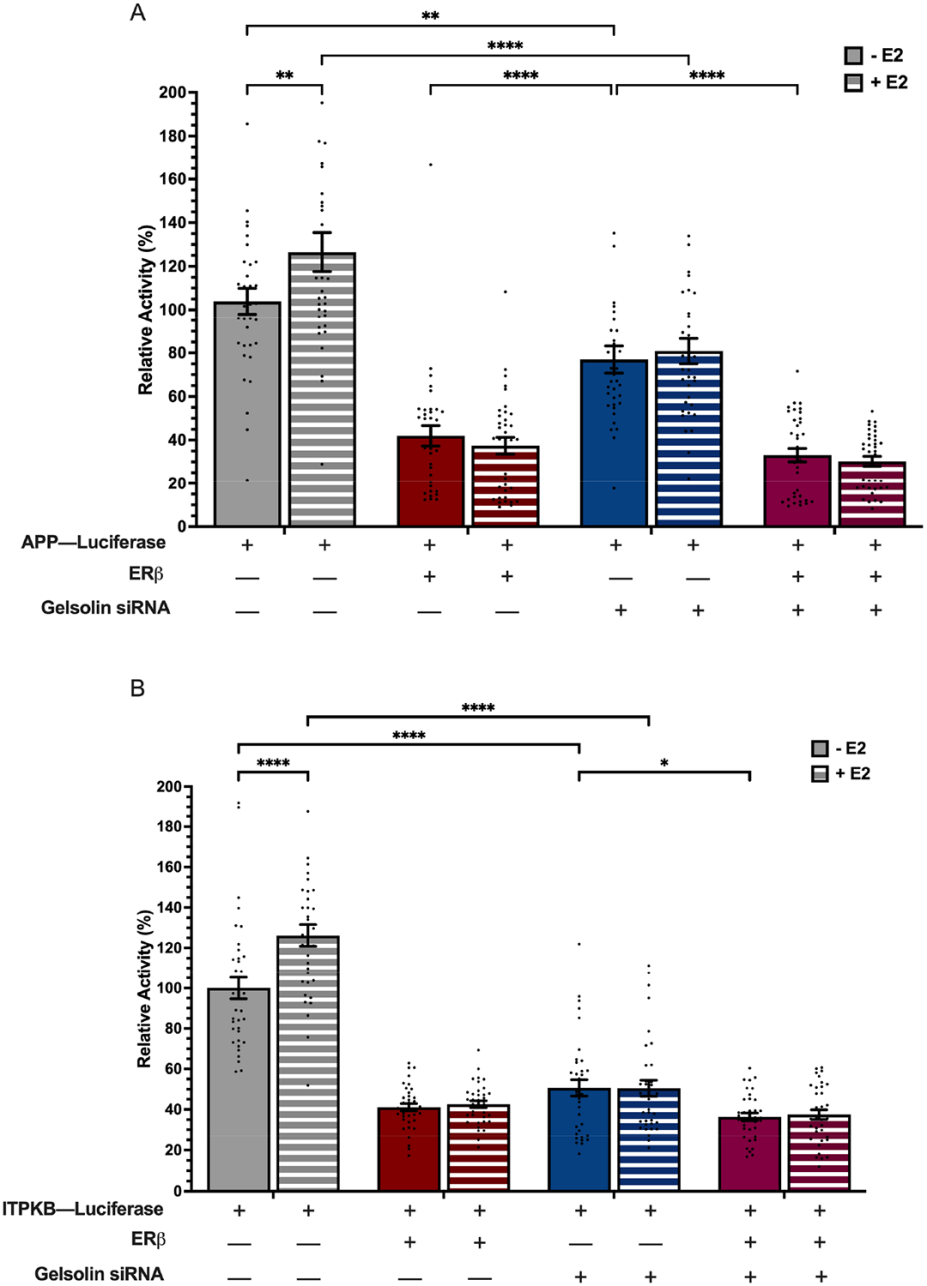
ERβ1 and GSN regulation of APP and ITPKB promoters is independent of E2. Luciferase activity was measured in SK-N-SH cells following transfection with human promoters for APP-luc (**A**) or ITPKB-luc (**B**), plus 20 ng ERβ1, siRNA-GSN and ±10 nM E2. Relative promoter activity was calculated based on % change from promoter-only group. Data sets are from 3 independent biological replicates with 12 technical replicates each. Data are depicted as mean ± SEM. An * denotes *p* < 0.05, **, *p* < 0.01, ****, *p* < 0.0001.

Our work, and that of others, has shown that ERβ1 can have ligand-independent effects in a variety of cell types [20-24]. Therefore, we next tested the effects of E2 treatment on ERβ1- and GSN-mediated regulation of APP and ITPKB promoters. Figure 4 shows that E2 treatment increased baseline levels of APP and ITPKB promoter activity (Figure 4A,B), demonstrating a previously unreported effect of E2 on APP and ITPKB gene regulation. However, E2 treatment had no effect on ERβ1-mediated repression of either promoter, and did not affect the observed decrease in promoter activity following GSN-siRNA knockdown.

### 3.6. ERβ1-Induced Repression of APP and ITPKB Promoters Likely Occurs Through Indirect DNA Binding

**Figure 5.**
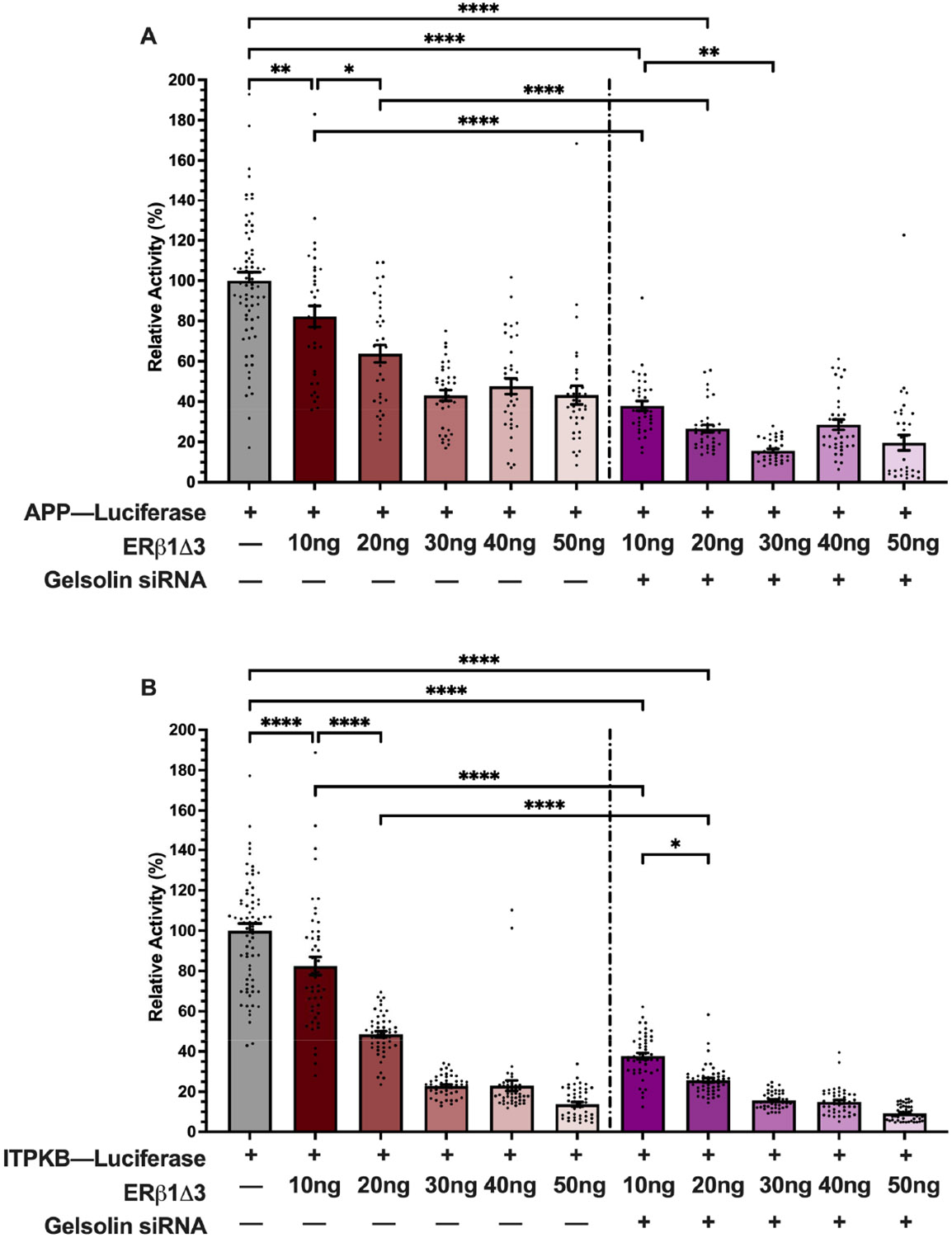
ERβ1Δ3 represses APP and ITPKB promoter activity. Luciferase activity was measured in SK-N-SH cells following transfection with human promoters for APP-luc (**A**) or ITPKB-luc (**B**), plus varying concentrations ERβ1Δ3 ± siRNA-GSN. Relative promoter activity was calculated based on percentage (%) change from promoter-only group. Data sets are from 3 independent biological replicates with 12 technical replicates each. Data are depicted as mean ± SEM. An * denotes *p* < 0.05, **, *p* < 0.01, ****, *p* < 0.0001.

ERβ1 binds directly to specific DNA sequence motifs or can be tethered to DNA as part of a larger regulatory complex. To test if ERβ1-mediated repression at the APP and ITPKB promoters was a result of direct ERβ1 binding or tethering, we took advantage of a structurally distinct, naturally occurring ERβ1 splice variant, ERβ1Δ3. The splice variant is endogenously expressed in the rodent brain [19] and is missing a significant portion of the DNA binding domain, including the second zinc finger. We predicted that if direct DNA binding to the promoters was required, then ERβ1Δ3 would not be able to repress APP and ITPKB activity. First, we transiently transfected varying concentrations of ERβ1Δ3 (10–50 ng) plasmid DNA with the APP-luc or ITPKB-luc reporter constructs. Our results showed that 10 ng of ERβ1Δ3 significantly repressed APP-luc promoter activity by 18% (Figure 5A). The APP-luc promoter activity was maximally repressed by 46% at 30 ng plasmid DNA and did not further decrease with higher concentrations of ERβ1Δ3 (Figure 5A). A similar result was observed with the ITPKB-luc promoter, except that it was maximally repressed by ~85% using 50 ng ERβ1Δ3 plasmid DNA (Figure 5B). These studies were performed in normal growth media and E2 treatment was not tested based on our earlier finding that ERβ-mediated repression of APP and ITPKB was not dependent on E2. The results shown in Figure 3 demonstrated that ERβ-mediated repression of APP and ITPKB was enhanced by GSN knockdown. Therefore, we next tested if ERβ1Δ3-mediated repression of the promoters was affected by GSN knockdown. Consistent with the results observed with ERβ1, ERβ1Δ3-mediated repression was further enhanced in the presence of GSN-siRNA.

## 4. Discussion

The goal of these studies was to determine the role of the ubiquitous cytoskeletal protein, GSN, in ERβ1 subcellular localization and function in neuronal cells. The results of our studies revealed three novel findings. First, GSN facilitated nuclear import of ERβ1 primarily in the absence of E2. The mechanisms for ERβ nuclear import in the absence of E2 have not been fully elucidated, and our results suggest that cytoskeletal proteins might play an important role in this process. Second, we demonstrate that ERβ1 repressed the promoter activity of two genes correlated with the severity and progression of Alzheimer Disease—amyloid-beta precursor protein (APP) and inositol–trisphosphate 3–kinase B (ITPKB). Several reports have shown that E2 regulates APP processing and turnover, but, to our knowledge, none have shown that the APP gene promoter can be directly regulated by an estrogen receptor. Further, there are no reports that ITPKB is regulated by estrogen receptors in any cell type. It is also likely that GSN independently increases APP and ITPKB promoter activity, as the activity of both promoters was significantly repressed when GSN was ~50% depleted in the cell. Finally, our data suggest that the ERβ1-mediated repression of APP and ITPKB is most likely due to ERβ tethering to other proteins, and not through direct DNA binding. Both of these promoters have multiple AP-1 motifs, and these results are in-line with our previous studies showing that ERβ1-induced repression at AP-1 motifs was abolished after GSN-siRNA knockdown [11].

Several cytoskeletal proteins are involved in the subcellular trafficking of steroid receptors [25,26], and our results are consistent with a previous study demonstrating that GSN facilitates nuclear import of the androgen receptor, another member of the nuclear receptor superfamily [12]. However, a unique feature of estrogen receptors is that they are primarily localized to the nucleus even in the absence of ligand binding [27-30]. Our data revealed that a siRNA-mediated reduction in GSN blocked the nuclear import of ERβ1 when E2 levels were low (i.e., in normal serum), but it had no impact when E2 levels were high (10 nM). These results suggest that GSN might be important for regulating the nuclear import of apo-ERβ1, which is critical for maintaining ERβ1-mediated transcriptional activity through dynamic hormonal fluctuations across the lifespan, such as menopause. Another interesting observation was that ERβ1 was diffusely distributed throughout the cell in media containing charcoal-stripped serum, which lacks endogenous hormones and most growth factors, suggesting that non-cognate ER ligands might be important for driving GSN-mediated translocation of ERβ1. Alternatively, evidence suggests that the subcellular distribution of ERβ1 is more heterogenous than ERα with nuclear localization dependent on neuronal subtype, brain region, and cytoplasmic organelles [28,30,31]. Notably, the nuclear localization of ERβ1 in media containing charcoal-stripped serum was completely restored by treatment with 10 nM E2 even when GSN levels were reduced. Collectively, our data reveal a complex interplay between GSN and the neuronal microenvironment for ensuring the nuclear retention of ERβ1.

The APP is a membrane-spanning protein that can be cleaved into multiple fragments. Under normal physiological conditions, APP plays a vital role in the central nervous system by interacting with cytoskeletal proteins to regulate cell and synaptic adhesion, axonal growth, and dendritic branching [32-38]. However, cleavage by β-secretase results in the formation of an insoluble Aβ_42_ fragment that aggregates as plaques in the extracellular space; a pathological feature that has long been associated with AD. Consistent with its neuroprotective effects, E2 increases the secreted form of Aβ, which is non-amyloidogenic and processed by α-secretase [39-41]. Several mechanisms are involved in the E2-mediated increase in soluble Aβ, including E2-stimulation of PKC to increase α-secretase activity, and the E2:ERα-mediated decrease in BACE1, the gene that encodes for β-secretase [41,42]. Our results provide evidence that ERβ1 adds another layer of regulation that is independent of E2, occurring at the level of APP transcription. Consequently, barriers that prevent ERβ1 nuclear retention with age, such as decreased association with GSN [11], and age-related declines in circulating E2, could hasten the overexpression of APP and cleavage of Aβ_42_ in postmenopausal women.

Inositol–trisphosphate 3–kinase B (ITPKB) is a major catalytic enzyme for inositol phosphates that regulate calcium handling in cells. ITPKB is ubiquitously expressed in most cell types including the brain, and its activity is calcium-dependent [43]. Overexpression of ITPKB, or the isoenzyme ITPKA, inhibits neurite outgrowth and is important for modulating dendritic spine structure through F-actin binding [44,45]. Importantly, ITPKB is overexpressed in the neurites of patients with AD and high levels are correlated with increased β-secretase and the accumulation of amyloid plaques [16]. To our knowledge, there have been no previous reports of estrogen receptor or E2 regulating the expression of ITPKB. Our data showing significant ERβ1-mediated repression of ITPKB promoter activity is consistent with a neuroprotective effect of ERβ1 and maintenance of synaptic plasticity. Moreover, GSN’s ability to bind and sever actin filaments is dependent on calcium, and GSN is significantly decreased in patients with AD [13]. Thus, the link between GSN, ITPKB:F-actin, APP, and Aβ plaques suggest a convergence of intracellular pathways that places ERβ at a critically important upstream functional node.

Multiple binding sites are predicted for ERβ1 in the APP and ITPKB promoters, including several putative AP-1 motifs (Eukaryotic Promoter Database; epd.expasy.org). The canonical AP-1 binding site (TGA(C/G)TCA) is recognized by heterodimers of the *FOS* and *JUN* family of proteins [46], and ERβ1 stimulates recruitment of this heterodimer to its target genes [2,3,47]. Our previous work showed that GSN knockdown abolished ERβ1-mediated repression at an AP-1 site, raising the possibility that GSN facilitates ERβ1 transcriptional activity [11]. Here, we show that GSN acts as a positive regulator of both APP and ITPKB promoters, as GSN knockdown significantly reduced their activity, even in the absence of ERβ1. The promoter activity was further repressed with ERβ1 + GSN-siRNA knockdown providing evidence that GSN and ERβ1 act cooperatively with other transcription factors, such as AP-1. This hypothesis is supported by our observation that the naturally occurring splice variant, ERβ1Δ3 was equally effective at repressing APP and ITPKB promoter activity indicating that direct DNA binding is not required. The alternative splicing of exon 3 eliminates one of the zinc fingers associated with the ERβ1 DNA binding domain; thus, this splice variant cannot bind to canonical estrogen response elements (ERE)s, but its ability to interact with other protein partners is not necessarily impacted [19]. However, we cannot rule out the possibility that ERβ1 and/or GSN act in concert with other transcription factors, such as SP-1, as these binding sites are also abundant in the APP and ITPKB promoters. Future studies are ongoing to determine the precise regulatory motifs required for ERβ1-mediated repression.

Notably, ERβ1 has been studied as a putative pharmacological target for AD treatment [48]. ERβ1-specific agonists, such as DPN, have demonstrated their capacity for mediating neuroprotection in aged animals and in mouse models of AD [49-51]. Moreover, long-term treatment of middle-aged rats with DPN revealed multiple gene targets of ERβ1 that collectively contribute to neuronal survival and synaptic plasticity [51]. Recently, a small clinical trial (27 patients) demonstrated that dietary supplementation with Genistein, a phytoestrogen with high ERβ1 affinity, improved cognitive performance in patients with prodermal AD [52]. It is important to note that although our study revealed that APP and ITPKB are novel direct targets for ERβ1 regulation, these studies were performed in an undifferentiated neuroblastoma cell line which might not be directly applicable to human pathology.

## 5. Conclusions

In conclusion, the results of these studies underscore the importance of cytoskeletal proteins, such as GSN, in regulating ERβ1 subcellular trafficking and transcriptional activity, especially in the absence of E2. GSN has been proposed to have a protective role in AD due to its ability to dissolve Aβ fibrils, and our data suggest an additional protective role through its facilitation of ERβ1 nuclear translocation. In addition, we provide evidence that ERβ1 represses the promoter activity of two genes correlated with progression and severity of AD, which highlights another previously unexplored avenue of ERβ1-mediated neuroprotection.

## Data Availability

The raw data supporting the conclusions of this article will be made available by the authors on request.

## Abbreviations

The following abbreviations are used in this manuscript:

AD: Alzheimer Disease
AP-1: Activator protein 1
APP: Amyloid beta Precursor Protein
E2: 17β-estradiol
ER, α, β1, β1Δ3: Estrogen receptor, alpha, beta1, beta1delta3
ERE: Estrogen response element
GSN: Gelsolin
HPRT: Hypoxanthine phosphoribosyltransferase 1
ITPKB: Inositol–trisphosphate 3–kinase B
siRNA: Short interfering ribonucleic acid
SK-N-SH: Human neuroblastoma cell line
